# A new ANMerge-based blood transcriptomic resource to support Alzheimer’s disease research

**DOI:** 10.1101/2025.10.02.25337067

**Published:** 2025-10-03

**Authors:** Nasim Mohamed Ismail, Maggie Miller, Hannah Crossland, Jalil-Ahmad Sharif, J Paul Chapple, Claes Wahlestedt, Kirill Shkura, Claude-Henry Volmar, Gregory Slabaugh, James A. Timmons

**Affiliations:** 1 School of Electronic Engineering and Computer Science, Queen Mary University of London, London, E1 4NS, UK; 2 Digital Environment Research Institute, Queen Mary University of London, London, E1 1HH, UK; 3 University of Miami Miller School of Medicine, Miami, Florida, FL 33136, USA; 4 Clinical, Metabolic and Molecular Physiology Research Group, School of Medicine, University of Nottingham, Derby, DE22 3DT, UK; 5 Faculty of Medicine and Dentistry, Queen Mary University of London, London, EC1M 6BQ, UK; 6 MSD Research and Development Innovation Centre, London, EC2M 6UR, UK.

**Keywords:** Alzheimer’s disease, Transcriptome, Blood, Machine Learning, Sexual dimorphism, Mitochondria, Multimodal, MRI

## Abstract

**INTRODUCTION:**

Alzheimer’s disease (AD) has greater prevalence in women and lacks effective treatments. Integrating multimodal data using machine learning (ML) may help improve diagnostics and prognostics.

**METHODS:**

We produced a large and updatable blood transcriptomic dataset (n=1021, with n=317 replicates). Technical robustness was assessed using sampling-at-random, batch adjustment and classification metrics. Transcriptomic and MRI features were concatenated to develop models for AD classification.

**RESULTS:**

Reprofiling of blood transcriptomics resolved previous technical artefacts (sampling-at-random AUC; Legacy=0.732 vs. New=0.567). AD-associated molecular pathways were influenced by cell counts and sex, including unchanged mitochondrial DNA-encoded RNA and altered B-cell receptor biology. Several genes linked to AD-associated neuroinflammatory pathways, including *BLNK*, *TREM2*, and *MS4A1*, showed significant enrichment. Concatenation of transcriptomics and MRI models modestly improved classification performance (AUC; MRI=0.922 vs. transcriptomics-MRI=0.930).

**DISCUSSION:**

We provide a new large-scale and technically robust blood AD transcriptomic dataset, highlighting details of molecular sexual dimorphism in AD and potential literature false positives, while providing a novel resource for future multimodal ML and genomic studies.

## 1 Introduction

Alzheimer’s disease (AD) is the most prevalent form of dementia, constituting ~60% of the estimated 55 million cases worldwide [1]. AD is characterised by progressive cognitive impairment, with definitive diagnosis confirmed postmortem by histopathological identification of Amyloid β (Aβ) plaques and hyperphosphorylated tau neurofibrillary tangles [2]. Importantly, it is widely accepted that AD begins many years prior to clinical diagnosis [3]. Research consortia such as the Alzheimer’s Disease Neuroimaging Initiative (ADNI) and AddNeuroMed have generated valuable multi-omics and neuroimaging resources (including magnetic resonance imaging (MRI)) to support the development of AD prognostics and diagnostics. Blood and cerebrospinal fluid (CSF) molecular profiles [4,5] have yielded several promising biomarkers for disease status [6,7]. For example, plasma phosphorylated tau^217^ (p-tau^217^) has demonstrated comparable performance to CSF p-tau^217^ for estimating brain Aβ and tau deposition using positron emission tomography (PET), offering a minimally invasive and cost-effective option [6]. It is understood, however, that the AD disease process is highly heterogeneous, with molecular features of resilience emerging [8], motivating the need for further genome-wide omics studies to explore the underlying molecular events, to better enable drug discovery.

Whole-blood transcriptomics is an attractive option for minimally invasive biomarker discovery, with early studies identifying potential pathological events occurring in the initial stages of disease, in addition to therapeutic targets [9]. Further, there is evidence that blood transcript profiling can track changes in cognitive status [10]. To date, the major biological pathway identified as differentially expressed in whole blood from AD patients is a reduction in mitochondrial nuclear-encoded oxidative phosphorylation (OXPHOS) gene expression [9,11], while preclinical and clinical studies have linked immune system dysfunction to AD pathogenesis [12]. Observational studies have also reported that low monocyte and eosinophil counts, and high leukocyte and neutrophil counts, are associated with increased risk of AD [13]. However, others have concluded that associations between neutrophil count and AD may be driven by confounding age-related variables [14,15]. Shifts in whole blood cell composition may complicate the interpretability of whole-blood transcriptome modelling, and it remains unclear whether reported molecular differences [9,11] are reliable features of AD. Critically, differences in gene expression signals between AD and control subjects driven by non-specific differences in cell populations may artificially inflate the performance of machine learning (ML) classifiers trained on AD blood transcriptomics or epigenomic data. The largest pre-existing transcriptomic datasets (from ADNI and AddNeuroMed/ANMerge) are limited by technical complications [16–18], including technical bias that specifically compromises their utility for building ML classifiers. Additionally, loss of the raw files prevents the ADNI probe signals from being re-extracted following validation against recent genomic databases. To date, these blood transcriptomics resources have also been limited in size to adequately assess the influence of sex; a critical consideration given the greater prevalence and distinct disease pathology of AD in women [19].

There is, therefore, a great need for larger, more robust, diverse, and updatable transcriptomic resources. Here, we introduce such a transcriptomic resource, built from whole-blood RNA samples from the AddNeuroMed (ANMerge) cohort. This new transcriptomic data is based on an array with 25-mer probes, which can be periodically realigned to the genome, to ensure that the data remains accurate over time. Further, we have established that this new data has reduced systematic bias, making it suitable for multimodal classification modelling. We revisit earlier reports that immune cell populations in whole blood do not influence pathway biology results [11], and illustrate that modelling variation in whole-blood cell composition does indeed influence the whole-blood molecular profile for AD. In doing so, we also identify molecular pathways that may be more sex specific. Finally, we illustrate how this new resource can be used to integrate transcriptomics and MRI features to develop prototype diagnostic models of AD status. Together, we provide a critical new resource suitable for classification studies, while our initial observations offer novel insights into the challenges of interpreting whole-blood molecular associations with AD.

## 2 Materials and Methods

### 2.1 AddNeuroMed transcriptomic cohort and newly derived Affymetrix GeneTitan transcriptomic dataset

The blood samples originate from subjects in the AddNeuroMed consortium, a large cross-European AD biomarker study, and the Dementia Case Register (DCR) cohort, and were provided to us by Dr Angela Hodges (King’s College London). They were processed in our laboratory to generate a new transcriptome resource. As per the original study protocol, subjects were excluded if they had neurological or psychiatric conditions other than AD, demonstrated a Geriatric Depression Scale score ≥ 4/5 or other unstable systemic illness. AD was diagnosed using the National Institute of Neurological and Communicative Disorders and Stroke and Alzheimer’s disease (NINCDS-ADRDA) and Diagnostic and Statistical Manual of Mental Disorders (DSM-IV) criteria for AD. Each subject underwent an interview and neuropsychological assessments (e.g. Mini Mental State Examination (MMSE)). Controls were also assessed using the CERAD battery [20]. Venous blood was collected into PAXgene^™^ tubes (Becton & Dickinson, Qiagen Inc., Valencia, CA), which were frozen at −20°C and then stored at −80°C. RNA was extracted using PAXgene^™^ Blood RNA Kit (Qiagen) according to the manufacturer’s instructions.

A total of 1,021 AD and control RNA samples were successfully profiled (passing standard quality checks), from 371 individuals with an AD diagnosis and 333 controls, and a further 317 technical replicates (from 149 AD and 115 controls). This new transcriptomic data was produced using the Affymetrix GeneTitan platform and an HTHGU133Plus PM array, following the manufacturer’s protocols (Karolinska Institute Core Facility, Huddinge, Sweden). As the genomic alignment of the 25-mer probes can be continuously updated, we realigned the probes to the GRCh38 and Gencode43 references, using methods previously described [21]. Briefly, a FASTA file representing the original array design was aligned against Grch38 - Gencode 43 [22] using the STAR aligner [23] and the probes with unique matches were retained. Probes with both a very low signal and a low coefficient of variation were removed, and the remainder were combined to form “probe-sets”, each with at least three probes (a process that yields a custom map file, known as a CDF, that can be updated routinely). Data was then normalised using iterative rank-order normalization (IRON) [24] in the default mode and subject to standard quality checks [21,25]. The custom CDF and new raw data are deposited at the Array Express (E-MTAB-15140), which contains up to 102,673 ENST labelled probe-sets (representing 21,045 genes). Of this, up to 59,493 probe-sets (filtered using absolute standard deviation values [21,25]) appear expressed in whole blood, of which there were 28,443 probe-sets with distinct signals per gene, reflecting a final total of 11,596 genes (as of May 2025, see supplementary data, Table S1). It is noteworthy that the ADNI consortium [6] no longer has access to the raw data (CEL files), preventing re-extraction of the data aligned to the current genome build, such that up to 30% of the ~2013 based annotations will be invalid.

A subset of the samples used to generate the new GeneTitan data was originally profiled using the Illumina Human HT-12 Expression BeadChip arrays. Unfortunately, these data (GSE63060 and GSE63061) suffer from batch effects of unknown technical origin. The data are therefore not suitable for building classification models, nor ideal for testing out hypothesis-based signature classifiers [16–18]. We refer to this original data as the “Illumina data” in the present article. We checked the validity of the Illumina HT12 V3 probes by aligning their sequences against GRCh38 - Gencode 43 [22] using STAR aligner [23] to confirm whether they remained valid or not (see supplementary methods). The same Illumina data has undergone reprocessing by the ANMerge team [4] and we also used this updated data source, comparing it with our reprocessing of the GSE63060 data (Figure S1). Reprocessing the original data yielded a greater number of transcripts (Illumina probes) than reported by the ANMerge portal data (See Table 1 for an overview of data resources). We refer to these processed datasets as “Illumina ANM” and “Illumina GSE63060”, respectively, in this article. The genes detected using the Illumina platforms are listed in Supplementary Data Table S1.

Whole blood is a mixture of cell types, with evidence of potentially coincidental or non-specific (for AD) shifts [13,26,27] in white blood cell populations between controls and AD subjects. Therefore, we applied cellular deconvolution [15,28] to estimate several white blood cell types in each sample, and studied how they may contribute to transcript differences between cases and controls. To adjust the data for any major technical factors, as well as model the influence of the white cell types, we used the ComBat package and supervised batch adjustment [29]. In this case, the IRON [24] normalised gene expression data were adjusted by total plate count (18 different 96-well plates contributed to the dataset) and the clinical site from which the samples were obtained. We refer to this adjusted dataset as the “whole blood” transcriptomic dataset hereafter. A third correction was applied to this “whole blood” transcriptomic data, adjusting for the variation in neutrophil count (which is by far the largest subcategory of blood cells, see Figure S2 and Results). We focused on neutrophils because we identified that they are strongly related to the global variance in gene expression, and they are the most numerous subtype of cell. We refer to this dataset as the ‘neutrophil-adjusted’ transcriptomic dataset hereafter.

### 2.2 Sampling ensemble gene sets to evaluate systematic bias

**Figure 1.**
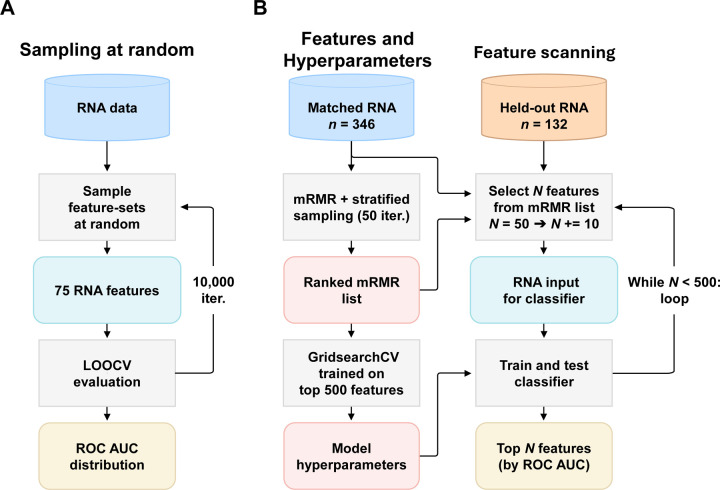
The workflow for evaluating systematic bias in AD and CTL blood transcriptome profiles and feature selection strategy. **A**: The workflow shows that 75 transcriptomics features were sampled at random from all available transcriptomics features in each data source. These features were used to train logistic regression classifiers for AD vs. CTL classification, using leave-one-out cross-validation (LOOCV). This process was repeated for 10,000 iterations to obtain a distribution of AUC scores. **B**: mRMR feature selection was applied 50 times on randomly selected 80% stratified subsamples of subjects from the matched transcriptomics data (n=346) to create 50 rank order mRMR lists of the top 500 transcriptomics features (‘genes’). The top 500 genes from each of the 50 mRMR analyses were ranked, and the average score for each gene was used to create a single overall rank. These top 500 features were then used as the input features for a grid search to acquire model hyperparameters. The feature list was also used to search for smaller subsets of features with higher performance in the AD vs. CTL classification task, by evaluating the performance of feature subsets from the top 50 to 500 genes, increasing in increments of 10 features. The final features selected were 200 or fewer features with the highest AUC obtained in the held-out test set (n=132 individuals with transcriptomic but no MRI data). Abbreviations: LOOCV, leave-one-out cross-validation; mRMR, minimum Redundancy Maximum Relevance; AD, Alzheimer’s disease; CTL, Control; AUC, area under the receiver operating characteristic curve; MRI, Magnetic Resonance Imaging.

Earlier work using the original AddNeuroMed Illumina data identified a batch effect of unknown origin between AD samples and control blood profiles [16], complicating interpretation of the data and rendering the data unsuitable for building machine learning classification models. The ANMerge consortium recently reported additional processing of this dataset to attempt to better control for this source of bias [16]. Therefore, we quantified systematic bias in these distinctly processed Illumina datasets, along with our new GeneTitan data, using a logistic regression classification method with default hyperparameters. Classifier performance was evaluated using a leave-one-out cross-validation (LOOCV) protocol, repeated over 10,000 iterations with gene sets of n=75 features sampled at random, each selected from the full dataset (Figure 1A and S1). This modelling approach quantifies signal inflation originating from dataset-specific biases that artificially improve classification statistics.

### 2.3 Differential gene expression and enrichment analysis

Differential gene expression (DE) analysis was performed using Significance Analysis of Microarray (SAM) [30] and all the available AD and control profiles, and focused on illustrating the influence of sex and white blood cell content on the biological processes attributed to AD (Input data reported in Table S2). Additionally, due to the limitations of using fixed statistical thresholds for studying the level of agreement between two analyses, we also used the RedRibbon R (RR) Package [31]. RR is a rank order method based on the hypergeometric distribution, and compares the rank order of the difference in gene expression signal in women (control vs. AD) with the difference in gene expression in men (control vs. AD). We applied multiple methods for pathway-level interpretation of differences in gene expression between AD and control samples, and the enrichment zone within the hypergeometric distribution plots produced by RR (See Results). Gene ontology [32] enrichment was calculated using the Metascape database [33] and the DAVID Database (Database for Annotation, Visualization, and Integrated Discovery (DAVID), https://david.ncifcrf.gov/) [32], with p-values generated versus the background of genes estimated to be expressed in our blood samples in both cases [34]. DAVID ‘GOTERM_BP_ALL’ results were processed and visualised using compareCluster [35] from the ClusterProfiler R package using this category. This approach enabled us to contrast the biological pathways associated with AD before and after adjustment for neutrophil counts, revealing the extent to which cell types may contribute to observed transcriptomic signatures. Furthermore, we examined gene expression patterns stratified by sex and, to our knowledge, report for the first time key differences in blood transcriptomic profiles between men and women with AD.

### 2.4 ANMerge MRI data with matching transcriptomics

ANMerge [4], a rebranded and updated version of the AddNeuroMed project, provides rich multimodal data suitable for integrative analysis. ANMerge includes data such as structural MRI, clinical data and several omics modalities, including genomics and plasma proteomics. This resource supports the development of multimodal ML models aimed at improving diagnostic accuracy by leveraging complementary information across data types. The present study adds a more robust and larger transcriptomics resource to enhance the ANMerge project.

To illustrate multimodal classification approaches, we used the structural MRI data acquired at the baseline visit for each subject available from the ANMerge portal and the new GeneTitan transcriptomics. The MRI data was acquired with 1.5 Tesla T1-weighted MRI protocols and is available as either raw MRI images or processed features. The processed features represent regional brain volumes and cortical thickness measurements for regions of interest (ROIs) computed using Freesurfer (version 6.0), a software package for analysing the functional, connectional, and structural properties of human brains using neuroimaging data [4,36]. All available MRI features (n=136) calculated using Freesurfer 6.0 were used to train MRI and transcriptomics-MRI models. Where multiple entry rows were present for a subject at their baseline visit, each MRI feature was represented by the mean value across all rows.

### 2.5 Data splitting and use

Transcriptomics data were available for 1021 AD and control transcriptomics samples, including technical replicates (from a total of 1,666 samples that were run, including individuals with a mild cognitive impairment (MCI) label, which are not reported in the present work). For the classification work itself, we excluded a further 57 samples with a slightly elevated normalised unscaled standard error, leaving 964 samples. We have not released the MCI samples due to the ambiguity in defining the true clinical status of each donor with respect to future AD status, and so the potential for misleading analysis. Among the 964 AD and control samples, there were 298 technical replicates, providing a further new and unique resource for future work. For evaluating multimodal classification models, a subset of 162 subjects with matched GeneTitan transcriptomic and Freesurfer MRI data was designated as the held-out test set. The remaining 504 samples were used to create a matched training set for transcriptomics models, and an additional held-out set for evaluating the performance of our feature selection steps (Figure 1B).

To limit the effect of the variables of sex and age on feature selection between AD and controls, propensity score matching [37] was used to create a matched subset of subjects for model training. Propensity score matching is a statistical technique that estimates the effect of a group difference by attempting to account for the covariates that may influence group assignment [37]. The Python package psmpy was used to calculate propensity logit values, using the clinical variables sex and age as covariates. Transcriptomics training samples (n=504) were matched using psmpy to create a class-balanced subset of subjects, matched on sex and age (n=346) [37]. Unmatched transcriptomics samples were then processed using propensity score matching to create a further class-balanced, transcriptomics-only held-out set (n=132). Summary statistics for the matched training set and transcriptomics-only held-out data are shown in Table 2.

### 2.5 Classification model feature selection

Transcriptomics features for model input were selected via a two-step feature selection process using the minimum Redundancy Maximum Relevance (mRMR) method [38]. We first applied mRMR to the matched training set to create a list of the top 500 features, which were both highly correlated with supervised classification labels and as independent as possible from each other, to reduce redundancy in the selection process. To minimise the effect of individual samples on mRMR feature selection, we employed a repeated subsampling approach where an 80% subsample of subjects, stratified for diagnostic class (AD or control) and sex (Female or Male), was drawn from the matched training set as input for mRMR, producing 50 ranked lists of the top 500 features per loop. Each transcriptomic feature was then assigned a rank value based on its placement across all lists. If a feature did not appear in one of the rank order lists, it was assigned a value of 0 for that list. The average rank value for each feature across all 50 rank order lists was calculated to create a final rank order. To identify an effective number of features to select for AD vs. control classification, we scanned the rank order to assess how adding additional features affects classification performance (Figure 1B). The top 50 features were first selected as input features, training classification models on the matched training set and then testing on the transcriptomics-only held-out test set. This process was repeated, adding 10 features each iteration until all 500 features were used (results are visualised in Figure S3). The subset of features which gave the highest Area Under the Receiver Operating Characteristic Curve (AUC) score was selected for testing on the final test set. To reduce model overfitting and ensure that both transcriptomics and MRI modalities had a similar number of features in multimodal fusion models, we chose the best subset of features with 200 or fewer features. If multiple feature subsets had the same AUC score, the subset with the smallest number of features was chosen. This two-stage selection strategy allowed us to balance relevance, non-redundancy, robustness to sample variability, and classifier performance, providing a well-grounded transcriptomic feature set for use in downstream modelling. We do not claim this is an exhaustive strategy for building an optimal transcriptomic classification model; just one sufficient for examining multimodal data integration and characterising the new AD community resource we present.

### 2.6 Model training and evaluation

When evaluating transcriptomics-only models, models were trained on the matched training set (n=346) and evaluated on the held-out test set (n=162), with hyperparameter tuning being performed on the training set (Figure 1B). For evaluating the performance of MRI and transcriptomics-MRI models, we used 50 repeats of 5-fold cross-validation, stratified for diagnostic class (AD or control) and sex (Female or Male) due to a lack of independent MRI data. For each training fold, hyperparameters were optimised exclusively on the training data using an exhaustive grid search with nested stratified cross-validation. Performance metrics are reported as AUC, sensitivity, specificity, accuracy in females, and accuracy in males. For cross-validated models, metrics were averaged across all test folds. SHAP (SHapley Additive exPlanations) [39] was used to illustrate how each input feature influences predictions from the top-performing transcriptomics-MRI models trained on whole blood and neutrophil-adjusted data. Shapley values were computed for all test samples across 50 repeats of stratified 5-fold cross-validation (162 samples per repeat). These values capture both the size and direction of each input feature’s contribution to the model’s predictions with respect to the AD class. Relative feature importance was determined based on the mean absolute Shapley value.

## 3 Results

### 3.1 Evaluation of bias in whole blood and neutrophil-adjusted transcriptomics data

**Figure 2.**
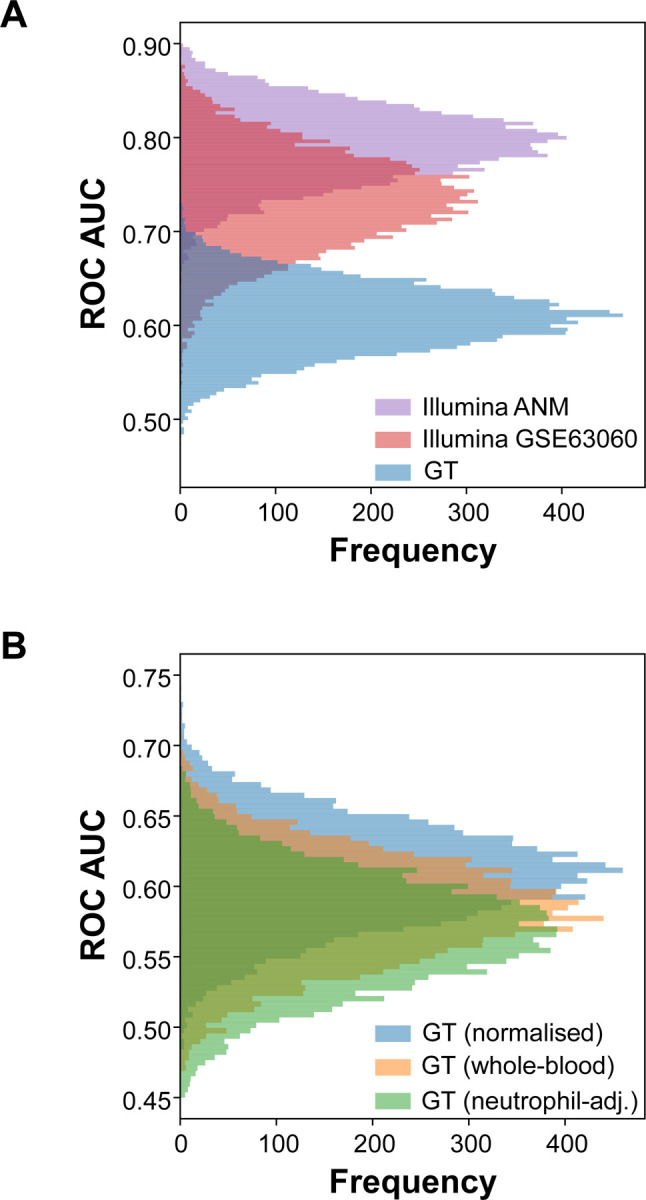
Evaluation of systematic bias in AD and CTL blood transcriptome profiles. Classifiers represented by 75 features were sampled at random from the ANMerge Illumina data source (Illumina ANM, n=162), an updated processing of the AddNeuroMed Illumina data (Illumina GSE63060, n=157) and a new Affymetrix GeneTitan transcriptomic data source (GeneTitan, n=346). Note that the GSE63060 raw data is from the same source data used by ANMerge, but was realigned and annotated to the latest genome in 2024. The third dataset was produced on an Affymetrix GeneTitan (GeneTitan) platform using an HTHGU133Plus PM array. **A**: The distribution of AUCs obtained from 10,000 iterations of sampling at random in the ANMerge reprocessed Illumina ANM data (purple), the original Illumina GSE63060 (red) data (reprocessed – see Methods) and the GeneTitan transcriptomic data (blue). **B**: Investigation of the influence of batch correction for technical variables and whole blood cell composition on the distribution of AUCs obtained over 10,000 iterations of classification using sampling at random; for the standard normalised GeneTitan transcriptomic data (blue), the GeneTitan transcriptomic data adjusted for two technical variables using COMBAT (plate total signal and clinical site (orange) and then this technically adjusted GeneTitan data adjusted for scaled neutrophil counts obtained from absolute immune signal deconvolution (green). Abbreviations: AD, Alzheimer’s disease; CTL, Control; AUC, area under the receiver operating characteristic curve.

To assess the suitability of our new transcriptomic dataset for use in ML studies, we evaluated the performance of randomly selected ‘gene sets’ for distinguishing between AD and control samples. If a dataset contains some form of bias (e.g. unexpected technical batches), then selecting sets of genes at random, to represent a classification signature, will yield a greater than expected classification performance (theoretically, a selected at random set of genes would produce an AUC=0.5, but this is unlikely due to the interconnected nature of the transcriptome). An elevated AUC can reflect confounding lab artefacts or study design, rather than biologically meaningful signal and thus limit the general utility of a dataset. The performance of a logistic regression classifier (any would be acceptable) was assessed using leave-one-out cross-validation repeated across 10,000 iterations, sampling 75 ‘gene sets’ at a time, is shown in Figure 2A. First, we compared the binary classification AUC scores for the standard normalised GeneTitan transcriptomic data (i.e. before any adjustment for key technical biases) versus the performance of the new ANMerge Illumina transcriptomic data, which was reported to have been reprocessed to reduce batch influences. Our analysis (Figure 2A) indicates that this Illumina data remains as, or more problematic, than previously identified [16,18] and should not be used in studies involving classification model development. In contrast, the GeneTitan data showed far less technical bias (Figure 2A).

We next examined the impact of adjusting gene expression data for selected technical variables (total plate signal and clinical centre), as well as the subsequent adjustment for estimated neutrophil counts (Figure 2B). Application of a step-wise COMBAT adjustment for plate signal, clinical centre and estimated neutrophil counts (Figure S2A and S2B) substantially removed the influence of these on the major sources of variation in the data. The technical adjustment reduced the mean classification performance of sampled at random gene sets from 0.608±0.04 to 0.584±0.04 AUC (Wilcoxon *P* value <0.001). Further, adjustment for variation in neutrophil counts led to a further reduction in classification bias (AUC=0.567±0.04 AUC, Wilcoxon *P* value <0.001). This highlights that any potentially non-specific shifts in whole-blood white cell subpopulations need to be carefully considered as they cannot be fully distinguished as being ‘disease-specific’ when building classification models. Further evidence of the robust technical performance of the new GeneTitan data was illustrated by considering the technical replicate performance (n=298), which demonstrated a global pairwise sample correlation of R=0.947±0.03, which was greater than the global pairwise sample correlation across all other samples (R=0.925±0.02).

### 3.2 Differential gene expression and enrichment analysis

Having established that our new large transcriptomic resource demonstrated substantially less bias in a classification setting than the existing ANMerge transcriptomic resource, we investigated the impact of cell composition and sexual dimorphism on AD-associated biological pathways. Some of our observations were striking and may have broader implications for the analysis of whole blood datasets [14,27]. The original analysis of Illumina data [11] used laboratory measurements of blood cell types. Based on a smaller sample size and conservative statistical methods, the authors reported that only basophils were significantly elevated in AD. However, given that basophils represent less than 3% of white blood cells, such a small shift in basophils would be unlikely to meaningfully affect global transcriptomic variation. Furthermore, they observed an approximately 5% increase in neutrophil count (see data plot in Figure 6A of Lunnon et al. [11], which uses a log_10_ scale). Neutrophil content, as identified using deconvolution [15,28], dominated global transcriptomic variation, which is unsurprising as neutrophils constitute over 50% of white blood cells in whole blood. Neutrophil count showed the strongest association with principal component 1 (PC1, R=0.45, *P* value <0.001) and PC2 (R=0.35, *P* value <0.001) – See Figure S2A. From a group mean perspective, only females had a higher estimated group mean neutrophil count (*P* value=0.0549), while males did not differ (Table S3). This highlights a potential sex-specific aspect of AD or may reflect poorer general health in women with AD compared to men.

**Figure 3.**
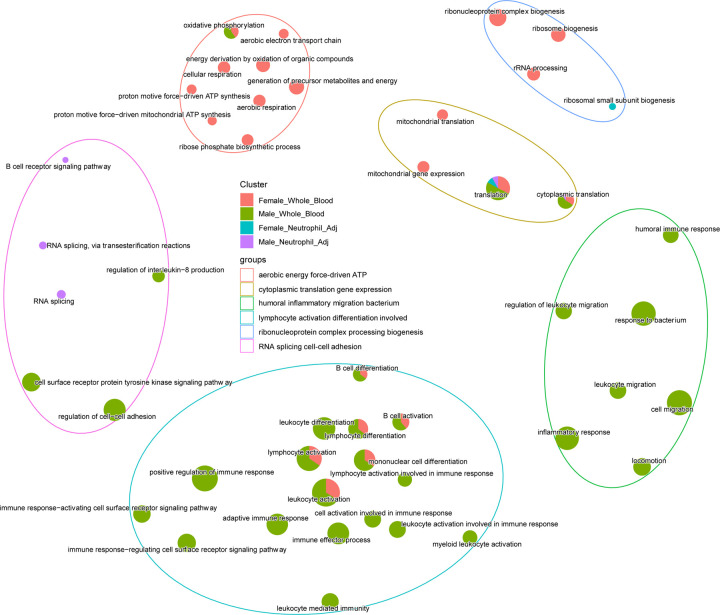
Integrating the biological pathways identified as regulated in AD, by sex (women (n=609) and men (n=412)) and following adjustment for the most abundant blood cell subtype, neutrophils. Within the ClusterProfiler package, the DAVID database was interrogated using each of the four lists of DE genes (using DAVID GOTERM_BP_ALL gene ontology categories). The significant ontologies (FDR<1%) for each list were visualised, and the inter-relationships across ontologies were grouped using the ClusterProfiler emapplot function (female whole blood (red), male whole blood (green), female neutrophil-adjusted blood (blue/cyan), and male neutrophil-adjusted blood (purple)). Abbreviations: DE, differentially expressed; DAVID, Database for Annotation, Visualization, and Integrated Discovery; Neutrophil_Adj, neutrophil-adjusted blood.

We found that the details of the biological pathways found by the DE analysis were largely dependent on how the data were processed (Table S4 and S5). This observation was independent of the particular pathway method used (Figure S4A and S4B). Contrasting whole blood and translation-related ribosomal genes were significant, regardless of data processing or sex (Figure 3, mustard-coloured grouping). In Figure S5A, we observed that the genes driving this consistent translation pathway enrichment (e.g. *MRPL3*, *MRPL35,* etc.) were downregulated in the whole blood data but showed slight upregulation after adjustment for neutrophil content. Notably, many downregulated genes were not subsequently upregulated after adjustment, so we cannot conclude that this result reflects a uniform shift, due to the signal processing methods.

Altered nuclear-encoded mitochondrial electron transport chain genes in AD (down-regulated) were also reported by Lunnon et al. [11]. In Figure S5B, we show a set of the nuclear-encoded OXPHOS genes obtained from the MitoCarta database (www.broadinstitute.org/) and observe a similar pattern. However, this mitochondrial pathway was differentially regulated in the opposite direction when neutrophil content was considered (Figure S5B), indicating that the precise associations with mitochondrial biology appear to be dependent on whether neutrophils are adjusted for or not. Previously, Lunnon et al. [9] used qPCR with a ribosomal house-keeping gene to report that mitochondrial DNA (mtDNA) encoded transcripts were *upregulated* in AD (in contrast with the nuclear-encoded DNA). They used qPCR as the Illumina array did not probe the mtDNA genes. The new GeneTitan data profiles six of the mtDNA and avoids assumptions about house-keeping genes, and we find that the mtDNA RNA expression was unaltered in all AD versus control comparisons (Figure S5C), suggesting that loss of ribosomal gene expression artifactually resulted in greater mtDNA gene expression when used as a house-keeping gene [9].

In whole blood, we observed that B-cell differentiation (Figure S4B) was an enriched pathway, while enrichment of B-cell receptor signalling was only detected in males (the genes driving these enrichments can be found in Table S6). An emerging contribution of viral infections to neurodegeneration has emerged in recent years [40], and this may contribute to the observed B-cell pathway enrichment. Consistent with greater evidence for altered B-cell biology in males, the Metascape analysis also identified modulation of B-cell biology exclusively in males, regardless of data processing (Figure S4B, Table S7). However, our analyses indicated that the female AD DE profile was characterised more by the broader term ‘immune response factors’, which may reflect the elevated group mean neutrophil count or reflect lack of specificity of the methods employed. Given the potential limitations of using fixed statistical thresholds for DE analysis, we used the RR hypergeometric distribution analysis (Figure S6, Table S8), which revealed a strong global consistency for DE between the sexes. The genes driving this shared relationship with AD status were involved in translation, mitochondrial biology, and RNA splicing (Figure S7). Notably, the directionality of gene expression differences within these common pathways depends on whether neutrophil content is adjusted for (Figure S5). It should also be noted that a majority of the DE between AD and controls is of a modest magnitude. Indeed, while Lunnon et al. [11] reported 2,908 DE probes (~2480 genes), only ~200 genes had a greater than 25% difference between groups (see Table S9). This in turn was before adjusting for any influence of the most abundant white blood cell types on differential expression, or consideration of the firmly established technical bias in that Illumina dataset (Figure 2A). In summary, many but not all pathway features of AD are consistent in men and women, but the nature of the identified relationship with AD depends on how white blood cell content is adjusted for, indicating that pathway enrichment statistics alone should not be used to interpret AD blood disease signatures.

### 3.3 Illustrating the utility of the data for multimodal classification

While interpretation of pathway biology is challenging in the face of alterations in white cell content, adjustment for technical factors and neutrophils reduces the bias between controls and AD samples. We next established the ability of whole blood RNA alone to distinguish between AD and controls using logistic regression, support vector machine and random forest classifier models, trained on whole blood and neutrophil-adjusted transcriptomic data. Across all three models (Table 3), we found that neutrophil-adjusted transcriptomic data had a reduced AUC when compared to whole blood models (Figure 2B). Random forest performed best across both formats of the GeneTitan transcriptomic data, achieving AUCs of 0.748 (whole blood) and 0.737 (neutrophil-adjusted). It is important to note that these results are illustrative and have not exhaustively explored or optimised models, and other approaches may yield superior performance; our focus is on characterising and releasing the data for the community.

To assess the potential of multimodal data integration, we combined transcriptomic and processed MRI features (calculated using Freesurfer 6.0, 136 features) using concatenation and compared classifier performance against the unimodal models. Model performance was estimated using 5-fold cross-validation repeated 50 times. Mean performance was calculated by averaging across all 250 tests (Table 4 and S10) and so will be optimistic compared with external validation (something that requires new AD cohort data to be generated). Multimodal support vector machine classifiers demonstrated the best performance in this analysis, achieving the highest AUC when trained on MRI concatenated with either whole blood or neutrophil-adjusted data (Table S10). Integrating transcriptomics with MRI data led to a modest performance increase (whole blood transcriptomic=0.935 vs. 0.922 AUC, Wilcoxon *P* value <0.001, and neutrophil-adjusted data=0.930 vs. 0.922 AUC, Wilcoxon *P* value <0.001). In line with findings from sampling-at-random (Figure 2B) and unimodal transcriptomics classifiers (Table 3), neutrophil-adjustment led to a lower AUC compared to whole-blood transcriptomics (0.930 vs. 0.935, Wilcoxon *P* value <0.001). This illustrates the potential for the systematic difference in cell counts between AD and control groups to act as a non-AD-specific bias [26,41,42]. Overall, these results highlight the potential value of integrating transcriptomic and MRI data for AD classification and the utility of the newly generated GeneTitan dataset to replace the ANMerge Illumina data for future multimodal classification studies.

### 3.4 SHAP feature importance analysis of best-performing transcriptomics + MRI fusion models

**Figure 4.**
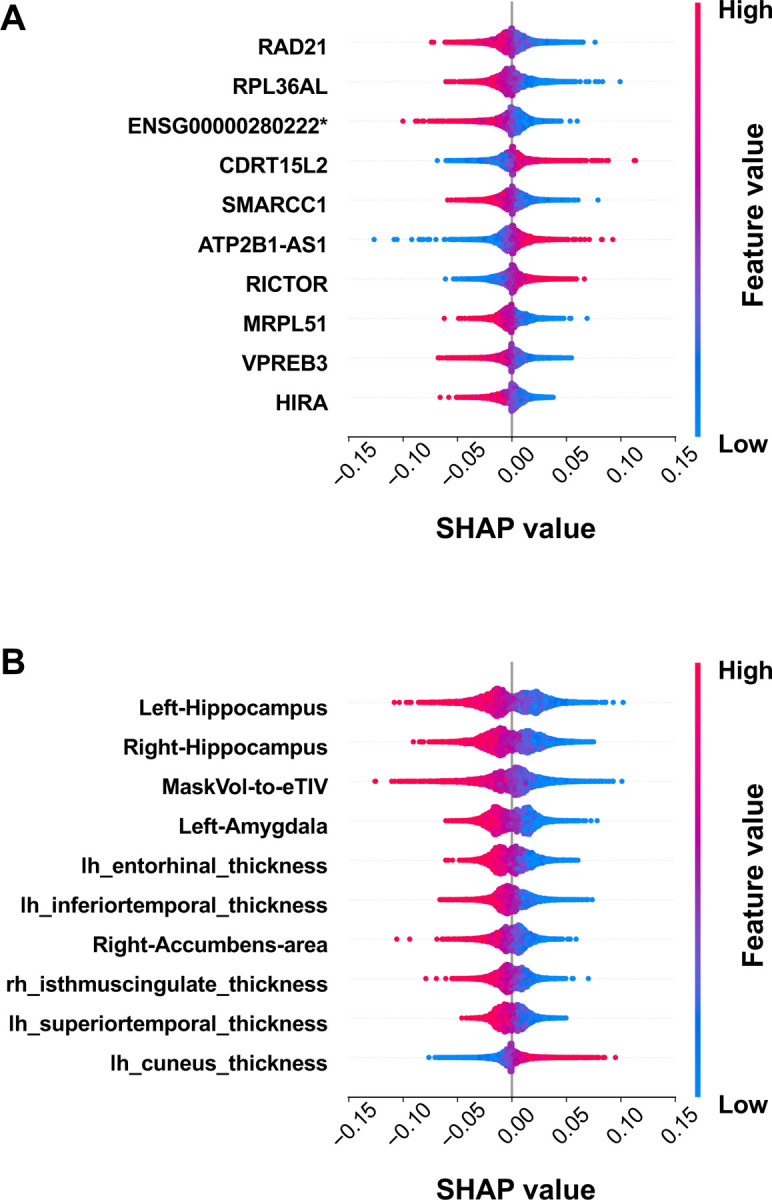
Assessment of the contribution of the top-ranked features to model performance. Shapley feature importance values were computed for input features for the best neutrophil-adjusted transcriptomics-MRI fusion models evaluated using 50 iterations of 5-fold stratified cross-validation (n=162; AD: 81, CTL: 81). Features are ordered by absolute mean Shapley value. Each dot represents a prediction, where a positive Shapley value indicates the feature is positively correlated with the AD class. Each dot’s colour represents the relative feature value. **A**: Dot plot of Shapley values for transcriptomics features. **B**: Dot plot of Shapley values for MRI features. *ENSG00000280222 is a TEC (To Be Experimentally Confirmed) transcript, not associated with a gene symbol at the time of analysis.

To visualise the feature-level contributions to the transcriptomics-MRI fusion model, we applied SHAP to models trained on neutrophil-adjusted transcriptomic data concatenated with MRI features. Figure 4 visualises the top 10 transcriptomic and top 10 MRI features, illustrating the direction of their influence on model predictions (magnitude of feature importances is illustrated in Figure S8). These features included several gene candidates that have been previously linked to aspects of AD biology in the literature (see Discussion).

## 4 Discussion

The original ANMerge blood transcriptomics has technical limitations [16,18] that make it unsuitable for building ML classification models, and these issues appear to be more pronounced in the updated data (Figure 2A). We present a new, large and more robust transcriptomic resource for the ANMerge project and one verified to be suitable for ML studies and matched with (Table S11) multi-modal data found in the ANMerge database. This new data is sufficiently large to study sexual dimorphism, while we illustrate that the influence of white blood cell content and sex on the molecular pathways associated with AD is complex. The new data relies on multiple dedicated 25-mer probes to quantify each transcript, and this allows the genomic alignment to be checked in future years and annotations to be updated. The ADNI data use a similar technology, but it cannot be updated [25] because the original raw data CEL files are no longer available. Previous conclusions, using the ANMerge cohort, that nuclear-encoded mitochondrial genes are suppressed while mtDNA-encoded genes are upregulated, do not hold true. While the new data provides an important new resource for the ML field, like most AD datasets, it was collected predominantly in white populations [43]. Recruitment of subjects from underrepresented populations remains a challenge [44], and their inclusion will introduce new confounders that must be considered when applying ML methods [45].

There remains a need for more diagnostic biomarkers which rely on minimally invasive methods. CSF-based biomarkers remain less than ideal as only 40% of individuals (e.g. in the UK) are willing to undertake a lumbar puncture, compared with 75% willing to have an MRI or PET scan, or 81% for blood tests, with women less willing to have a lumbar puncture than men (37% of women vs. 48% of men) [46]. Recent studies show that plasma p-tau^217^ demonstrates diagnostic accuracy equivalent to CSF p-tau^217^, indicating that plasma-based markers can act as a viable alternative to CSF markers in detecting AD pathology. Meta-analysis found that plasma p-tau^217^ had comparable sensitivity and specificity for detecting Aβ and tau PET deposition to CSF p-tau^217^ [7]. Elevated levels of plasma p-tau^181^ were associated with poorer neuropsychological test performance based on retrospective analysis of subjects from the ADNI dataset [6]. However, these biomarkers are unable to provide novel information on disease pathology at the genome scale, and so omics profiling of blood samples remains an important objective.

Multimodal learning has been used to enhance performance in disease classification and progression prediction tasks [47,48]. In the present study, using concatenation, a simple fusion approach [49], provided only a modest improvement in classification performance, compared to unimodal models trained on MRI features (Table 4). The relationship between MRI features and Illumina transcriptomics was previously explored, where linear regression was used to assess associations between a PCA-based summary value for gene co-expression modules and structural MRI features in the AddNeuroMed cohort. They reported a significant positive correlation between the eigengene value of a mitochondrial (OXPHOS) enriched large module and hippocampal volume measures. Whether this association remains after correction for alterations in blood cell composition (Figure 3) and the absence of explicit technical issues remains unknown. Alternate methods of feature representation may enhance learning. For example, SurvPath [50] used graph-based representations of transcriptomic features representing cancer pathways to generate 331 pathway-based tokens, reducing the dimensionality of transcriptomic data. These were then combined with whole-slide image embeddings to train deep early fusion models. Incorporating strategies for improving multimodal learning could lead to identifying transcriptomics features (or groups of them) more diagnostic of AD by capturing interactions between transcriptomics and MRI data [49]. Like our analysis, Maddalena et al. [51] used MRI and transcriptomics, and reported that fusion by concatenation improved classification performance using the Illumina ANM data. It is probable that the substantial improvement in classification reported reflects bias in the transcriptomic data (Figure 2A and 2B), as we do not find a similar level of improvement with the methods used in the present study.

Multimodal AD datasets containing both imaging and genetic data have also been leveraged to train supervised ML models. Zhou et al. [47] used integrated gradients on a deep fusion AD classifier trained on MRI, PET, and genetic data from the ADNI cohort to identify the most influential ROIs and single-nucleotide polymorphisms for driving classifier predictions. The top MRI features identified in our study (Figure 4B and S8B) represented the hippocampus, normalised brain mask volume (MaskVol-to-eTIV), and the amygdala. MaskVol-to-eTIV normalises brain region volumes (MaskVol) by estimated intracranial volume (eTIV), but in AD, brain atrophy can bias detection of eTIV estimates downward, affecting the accuracy of this ratio [52]. Amygdala atrophy observed in MRI has been associated with downstream cognitive impairment [53]. Hippocampal and Amygdala features also scored highly in SHAP feature importance analysis of random forest models trained on Freesurfer MRI features from the ADNI cohort by Song et al. [54]. Nevertheless, further consideration of how the MRI data is processed is also merited when attempting new multimodal classification models with these data.

One of the striking observations in our study is the influence of cell proportions on the pathway biology, particularly neutrophils, the major component (typically 40–60%) of leukocytes in peripheral blood [55]. While correction for immune cell type unambiguously reduces the performance of ‘random’ transcriptomics classifiers, it complicates the interpretation of AD pathway biology. Previous studies have pointed to a mechanistic role of inflammation in AD, with one notable study reporting that individuals with chronic and/or increased proinflammatory cytokines over time tend to have the most dramatic cognitive decline [56]. Neutrophil-to-lymphocyte ratio (NLR) is considered a crude marker of inflammatory status in cancer, cardiovascular and inflammatory diseases [57,58]. Elevated levels of neutrophils have been observed in neurodegenerative diseases such as PD and chronic conditions prevalent in AD populations, such as Type 2 Diabetes [42,59]. NLR has been investigated in AD, where it was initially found to be correlated with amyloid burden in the AIBL cohort but not significantly, after correction for age, sex, and APOE ε4 allele status [14]. Typically, AD study protocols control for various conditions during study recruitment, but this will not be the situation in the real-world application of any new diagnostic. Beyond their role as potential confounders, neutrophils may have direct involvement in AD pathogenesis. Recent evidence suggests neutrophils infiltrate the brain in AD, migrating toward amyloid plaques and contributing to blood-brain barrier disruption, capillary blood flow stalling, and neuroinflammation through mechanisms including neutrophil extracellular trap (NET) formation [60,61]. Clinical studies have demonstrated neutrophil hyperactivation in AD patients, with increased reactive oxygen species production, elevated intravascular NETs, and altered neutrophil phenotypes that correlate with disease progression [62]. NETs have been directly observed in both cortical blood vessels and brain parenchyma of AD patients, suggesting a mechanistic role in vascular and neural damage [60]. However, our statistical adjustment for neutrophil proportions as a potential confounder does not preclude their mechanistic involvement in disease pathogenesis. Through consideration of the influence of white blood cell content, we illustrate the potential impact on the specificity of any diagnostic for AD, rather than evaluating what their role, if any, is in AD pathophysiology.

Consequently, we find that previously reported deficits in nuclear-encoded mitochondrial OXPHOS gene expression, a reliable feature of human neuromuscular ageing [63], are not a robust feature of AD in blood. There was also evidence for a greater prevalence of inflammatory pathways modulation in women with AD, which could be the result of oestrogen depletion that occurs post-menopause. Post-menopausal women display higher levels of pro-inflammatory cytokines, most strikingly IL-6, which is a potent mediator of immune response that we also see elevated in AD individuals [64–66]. Our observation of sex-specific modulation of inflammatory pathways supports the role of sex-specific mechanisms in Alzheimer’s disease, which may in part account for the nearly doubled lifetime risk of AD in women compared with men (1 in 5 versus 1 in 10 at age 45) [67].

From our analysis in men, we identified immune genes upregulated after correction for neutrophil count (Table S5), several of which are implicated in dysregulation of microglial function (*BLNK*) [68], calcium signalling (*MS4A1*) [69] and inflammasome dysregulation (*CARD16*) [70,71]. Microglial function has long been studied in relation to AD because of the ample evidence suggesting that neuroinflammation contributes to disease progression. It is theorised that microglia enter an activated state in response to Aβ, which then release pro-inflammatory cytokines. The chronic activation of microglia reduces their ability to clear Aβ and disrupts the balance of anti-inflammatory and pro-inflammatory signalling. The buildup of both Aβ and pro-inflammatory cytokines leads to the activation of more microglia, resulting in exacerbated neuroinflammation and eventual neurodegeneration[72]. *BLNK* encodes a B cell linker protein, which is a molecular driver of the transition of AD-associated microglial subtypes [68]. It participates in the recruitment of PLCG2, which is an integral part of the *TREM2* signalling cascade [68,73]. *TREM2*, essential for microglial activation, metabolism, phagocytosis, CNS immune response, and overall brain homeostasis, has an established role in AD progression [74,75], with AD patients displaying increased TREM2 levels [76,77]. Since *BLNK* interacts with the *PLCG2* and the *TREM2* signalling pathways, our finding of *BLNK* upregulation fits with previous findings of upregulated *TREM2* in AD individuals.

Calcium signalling is essential for neurotransmitter transmission, synaptic contact, cell proliferation, and apoptosis, so it is therefore unsurprising that alterations in calcium homeostasis are prevalent in AD individuals [78]. The *MS4A1* gene, which we found upregulated in AD men after neutrophil adjustment, encodes a B-cell surface marker, CD20. *MS4A1* is involved in calcium conductance and promotes calcium influx via channel opening [79]. *CARD16*, a member of the caspase activation and recruitment domain (CARD) family, promotes neuroinflammation via caspase-1 activation, which leads to IL-1β release [71]. Heightened IL-1β levels have been reported in both the CNS and periphery of AD patients compared to healthy controls [79,80], making it biologically plausible that we observe an upregulation of CARD16 in men with AD.

We found specific evidence for B-cell modulation in men, an unsurprising finding as B-cells are responsible for antibody production and therefore a key component of the adaptive immune response. Identified genes were directly involved in the B-cell receptor signalling cascade (*CD79A*, *BLNK*) or regulation of B-cell activation (*BANK1*) [81,82]. In triple transgenic models of AD, B-cells are implicated in both reducing Aβ plaques and accelerating neuroinflammation, with the specific subtypes of B-cells being critical [83]. Depletion of B-cells also worsened spatial learning and memory defects in AD mice, which was also associated with increased Aβ burden [84]. Conversely, B-cell-deficient mice have elevated IgG around Aβ plaques and increased activation of microglial cells, with further B-cell depletion resulting in reduced amyloid deposits and improved cognitive function [85]. Thus, B-cells could have a neuroprotective function in early AD pathology, proposed to be related to the production of the ‘anti-inflammatory’ cytokine interleukin-35 [84]. Nevertheless, given the complexities of correcting for differences in cell populations, caution is required when interpreting pathway-level observations applied to transcriptional analysis of whole blood.

The top ten transcriptomics features identified in the best neutrophil-adjusted transcriptomics-MRI model using absolute mean Shapley values (Figure 4A and S8A) also included genes involved in several AD-associated disease mechanisms, including neuroinflammation [86], metabolic dysfunction [87], synaptic dysregulation [88–90] and epigenetic dysregulation [91]. *RAD21* is a transcriptional regulator which promotes M2 microglial polarisation, reducing neuroinflammation and neuronal loss in AD mice [86]. *RICTOR* encodes a subunit of the mTORC2 complex and mTOR signalling is down regulated in normal neuromuscular ageing and activated in AD [63], potentially contributing to dysregulation of energy metabolism, synaptic plasticity, and autophagy [87]. *SMARCC1* encodes a subunit of the SWI/SNF complex, which modulates DNA-nucleosome interactions to regulate transcription of genes for neurodevelopment, cell cycle regulation, and differentiation [88]. *HIRA*, a histone chaperone [89], was also identified as negatively associated with the AD class. Deficits in *HIRA* could contribute to AD pathology through epigenomic dysregulation, leading to silencing neuroprotective genes, promoting neuroinflammation [89,90]. These observations provide support that the transcriptomic classification signature relies on genes with known or putative roles in AD disease pathways, although further independent validation is needed to confirm the classification-based observations.

## Supplementary Material

Supplement 1

Supplement 2

Supplement 3

Supplement 4

## Figures and Tables

**Table 1.** Summary statistics for samples available in the new Affymetrix GeneTitan transcriptomic dataset (GeneTitan, n = 1021) and normalised Illumina HT12 V3 + V4 transcriptomics from the ANMerge dataset (Illumina ANM, n = 691).

Feature	GeneTitan	Illumina ANM
**Samples**	1021	511
**AD**	567	275
**CTL**	454	236
**Female/Male, %**	59.65%	62.21%
**Age, mean**	75.77	75.14
**Age, range**	[41,95]	[52,88]
**Genes**	21015	5212*

Note: n = 1021 GeneTitan profiles passed QC checks and are reported here and deposited at ArrayAssist (E-MTAB-15140). AD and CTL counts are based on the final diagnosis clinical labels.

* : Number of genes available in normalised Illumina ANM data. In our study, we also realigned and annotated GSE63060 (AddNeuroMed batch 1 Illumina HT12 V3, deposited in Gene Expression Omnibus) raw data, which is largely identical as it comes from the same laboratory files used by ANMerge, to the latest genome in 2024 to acquire data representing 12061 genes. Abbreviations: AD, Alzheimer’s disease; CTL, Control.

**Table 2.** Summary statistics for subjects in matched training, transcriptomics-only held-out and transcriptomics + MRI held-out sets.

	Training RNA-only (346)		Held-out RNA-only (132)		Held-out RNA+MRI (162)	
**Diagnosis**	AD (173)	CTL (173)	AD (66)	CTL (66)	AD (81)	CTL (81)
**Sex, n (%)**						
**Female**	100 (57.80%)	100 (57.80%)	43 (65.15%)	53 (80.30%)	55 (67.90%)	49 (60.49%)
**Male**	73 (42.20%)	73 (42.20%)	23 (34.85%)	13 (19.70%)	26 (32.10%)	32 (39.51%)
**Age, years**						
**Mean (SD)**	76.24 (6.70)	76.24 (6.70)	76.70 (11.40)	71.50 (7.91)	75.63 (7.01)	72.28 (6.42)
**Range**	[56,92]	[56,92]	[50,95]	[52,92]	[58,89]	[55,88]

Note: Held-out RNA + MRI was used as a test set to evaluate transcriptomics-only models trained on training data and as a cross-validation set for transcriptomics-MRI fusion models. Abbreviations: RNA, Transcriptomics; AD, MRI, Magnetic resonance imaging features extracted using Freesurfer 6.0; Alzheimer’s disease; CTL, Control; SD, standard deviation.

**Table 3.** AD vs. CTL classification performance of whole blood and neutrophil-adjusted transcriptomics evaluated on logistic regression, support vector machine, and random forest models.

Dataset	Model	Features	AUC	Sens.	Spec.	Acc. (F)	Acc. (M)
Whole blood	Logistic regression	110	0.706	0.728	0.556	0.702	0.534
Whole blood	Support vector machine	110	0.737	0.753	0.580	0.712	0.586
Whole blood	Random forest	150	0.748	0.716	0.630	0.692	0.638
Neutrophil-adjusted	Logistic regression	120	0.691	0.704	0.679	0.721	0.638
Neutrophil-adjusted	Support vector machine	120	0.724	0.679	0.667	0.692	0.638
Neutrophil-adjusted	Random forest	160	0.731	0.716	0.630	0.683	0.655

Note: Models were trained on data for 346 subjects (AD, n = 173; CTL, n = 173) and evaluated on 162 subjects (AD, n = 81; CTL, n = 81). Abbreviations: AUC, Area Under the Receiver Operating Characteristic Curve; Sens., sensitivity; Spec., specificity.; Acc. (F), accuracy for female subjects; Acc. (M), accuracy for male subjects

**Table 4.** AD vs. CTL classification performance of multimodal models integrating whole blood and neutrophil-adjusted transcriptomics with MRI data evaluated on support vector machine models.

Multimodal	Model	AUC	Sens.	Spec.	Acc. (F)	Acc. (M)
No	RNA (Whole blood)	0.733	0.726	0.619	0.663	0.687
No	RNA (Neutrophil-adjusted)	0.704	0.730	0.620	0.658	0.703
No	MRI	0.922	0.823	0.867	0.836	0.862
Yes	RNA + MRI (Whole blood)	0.935	0.841	0.854	0.828	0.881
Yes	RNA + MRI (Neutrophil-adjusted)	0.930	0.839	0.856	0.833	0.874

Note: Models were evaluated using 50x repeated 5-fold cross-validation on 162 subjects (AD, n = 81; CTL, n = 81). Abbreviations: AUC, Area Under the Receiver Operating Characteristic Curve; Sens., sensitivity; Spec., specificity; Acc. (F), accuracy for female subjects; Acc. (M), accuracy for male subjects.

## Data Availability

The raw gene expression data reported in this paper will be available at E-MTAB-15140, along with the processed data files. The probes for the raw data can be realigned to the current genome and transcriptome each year to remain current. Code for the various informatics analyses can be readily obtained by contacting the authors or via https://github.com/Nasim-MI/Affy-ANMerge-ML.
