# Supplementary material for: A new ANMerge-based blood transcriptomic resource to support Alzheimer’s disease research
Source: medRxiv. 2025 Oct 3:2025.10.02.25337067. Preprint. [Version 1] doi: 10.1101/2025.10.02.25337067 (PMC12622077; doi:10.1101/2025.10.02.25337067)
Supplement: Supplement 1 [file media-1.docx]

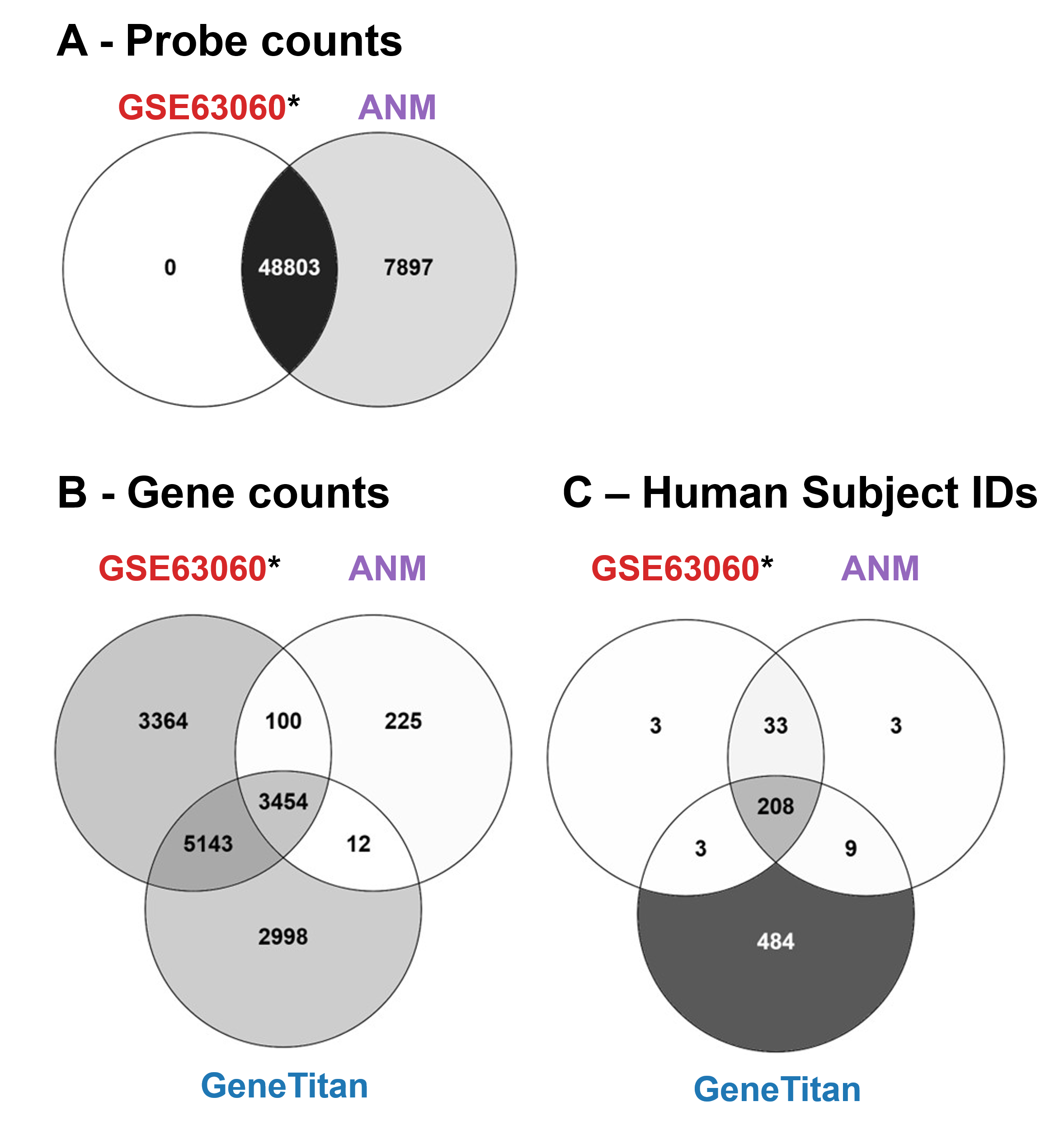


**Figure S1.** GeneTitan Metrics for probes or genes (rows of data) per data set. *GSE63060 raw data is largely identical as it comes from the same laboratory files used by ANMerge, but was realigned and annotated to the latest genome in 2024. The third data set was produced on an Affymetrix GeneTitan (GeneTitan) using an HTHGU133Plus PM array. **A:** Venn diagrams comparing Illumina probes in GSE63060 after full processing and realignment to the current genome/transcriptome versus the Illumina batch 1 data deposited by ANMerge (ANM). **B:** Ensembl gene IDs (ENSG) present after processing and realignment to the current genome/transcriptome processed in the GSE6030 data, the ANM data and new Affymetrix GeneTitan data (also processed and re-aligned to the current genome) and **C:** Total number of AD and CTL subjects (Final Diagnosis labels) available in each data set and the overlap in membership across the three sources of data (GSE63060: n=247, ANM: n=253 and Affymetrix: n=704 individuals (1021 arrays in total as we produced technical duplicates)). Note that ANMerge contains additional transcriptomic data from AddNeuroMed batch 2 (unnormalized AD + CTL, n=253), but with far fewer accompanying MRI scans (n=7).


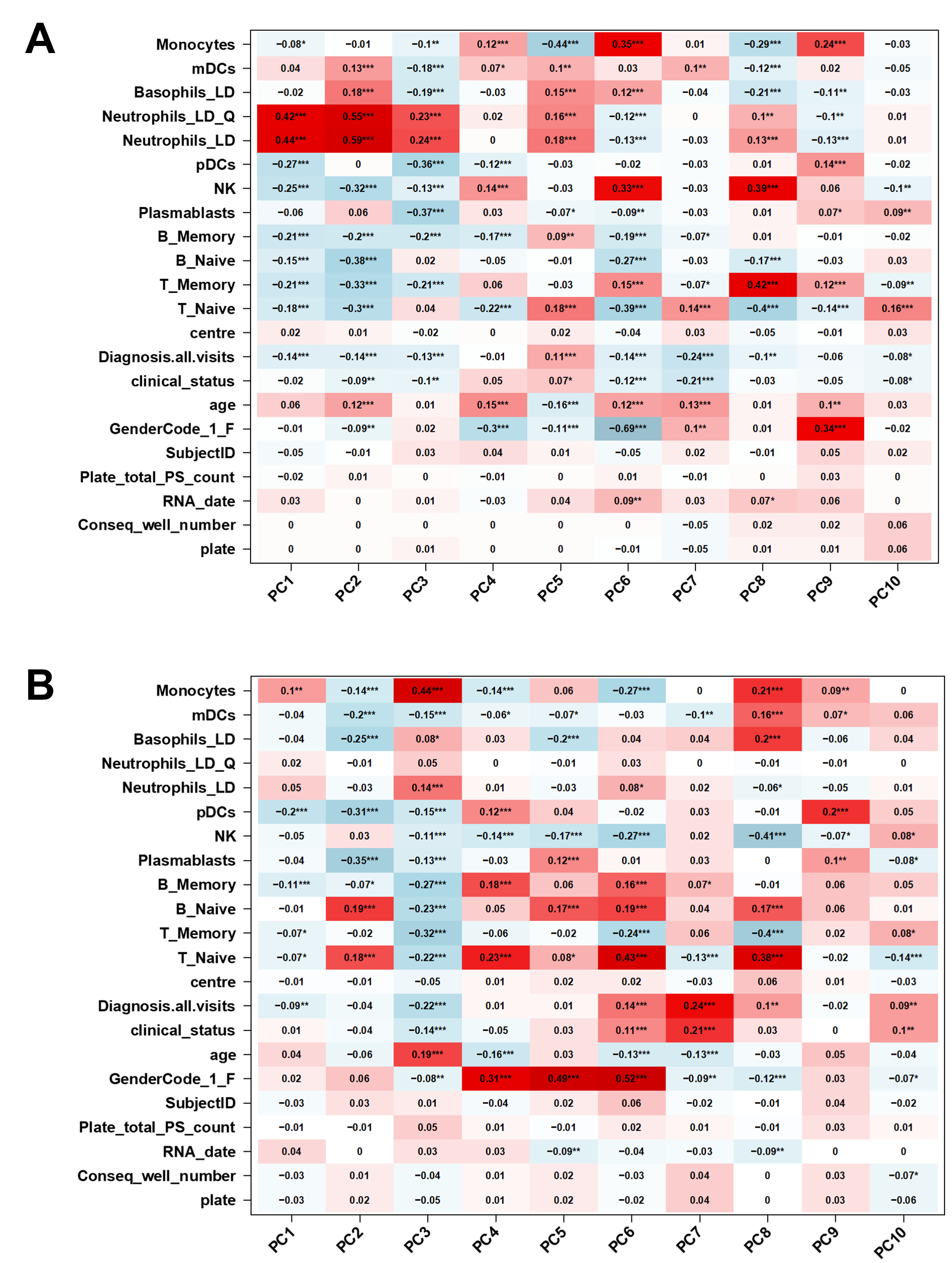


**Figure S2**. Principal component (PC) analysis of lab and biological variables in Affymetrix GeneTitan transcriptomics dataset (N = 1021). Asterisks indicate levels of statistical significance: *P* value < 0.05 (*), *P* value < 0.01 (**), *P* value < 0.001 (***). Colour gradient ranges from blue, indicating strong negative values, to red, indicating strong positive values. **A:** PC analysis in the whole blood data (corrected for plate total signal and clinical centre adjusted data). Note that Age, Diagnosis and Sex are still clearly visible as related to PCs. **B:** PC analysis after combat adjustment for variation in neutrophil quartiles limits the influence of neutrophil count on variation in gene expression. Note that estimated neutrophil counts covary with other cell types due to the deconvolution method (which we scale to a total cell count of 100).


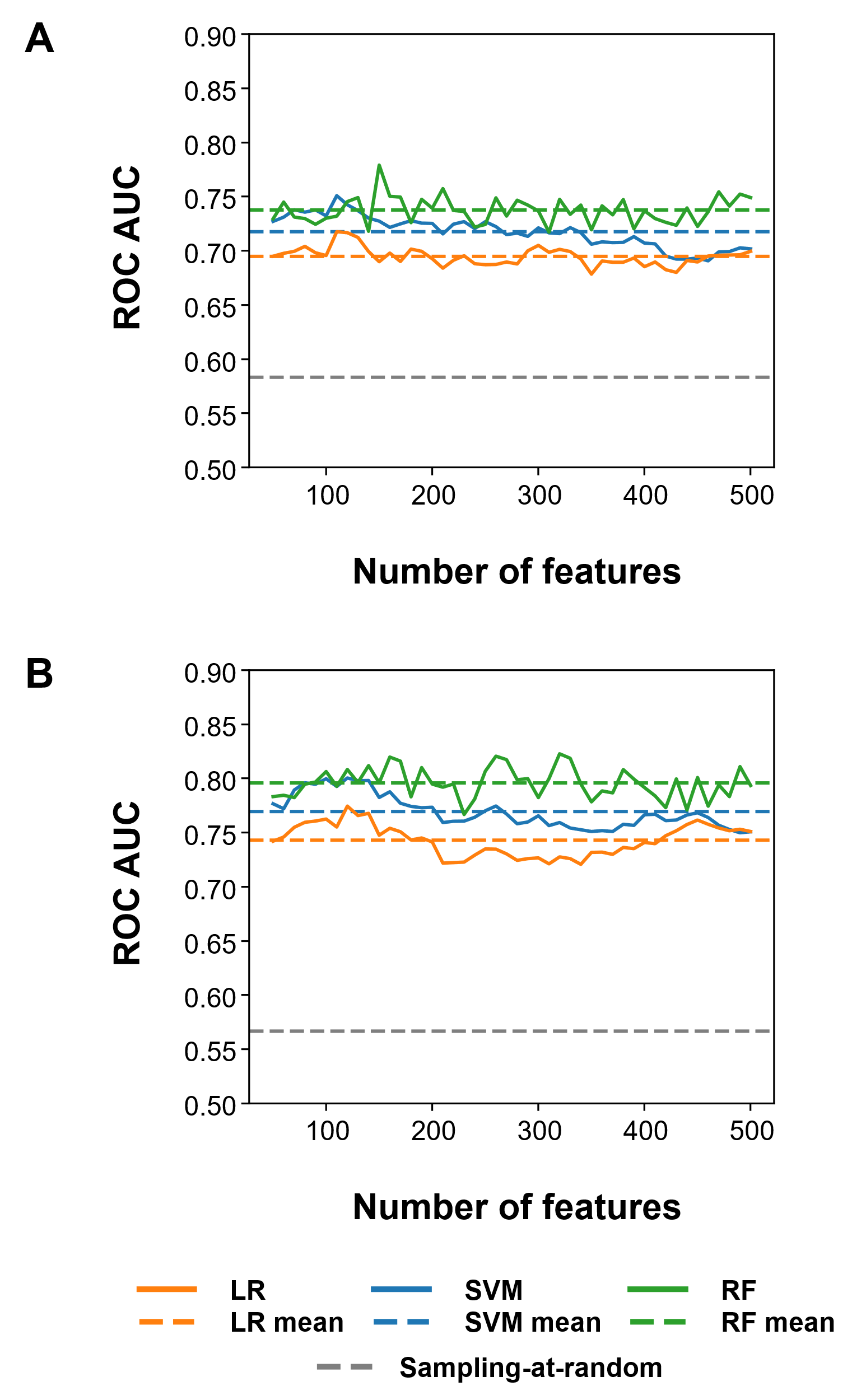


**Figure S3.** Scanning across the top 50-500 features, increasing in steps by 10 features from subsampled mRMR rank order and evaluating transcriptomics classifiers (training set, n = 346; validation set, n = 132). Mean classifier AUC from sampling-at-random for each mean dataset (see Figure 2B) is also shown for comparison (grey). **A:** Whole blood data. **B:** Neutrophil-adjusted data. Abbreviations: LR, Logistic regression; SVM, Support Vector Machine; RF, Random forest.


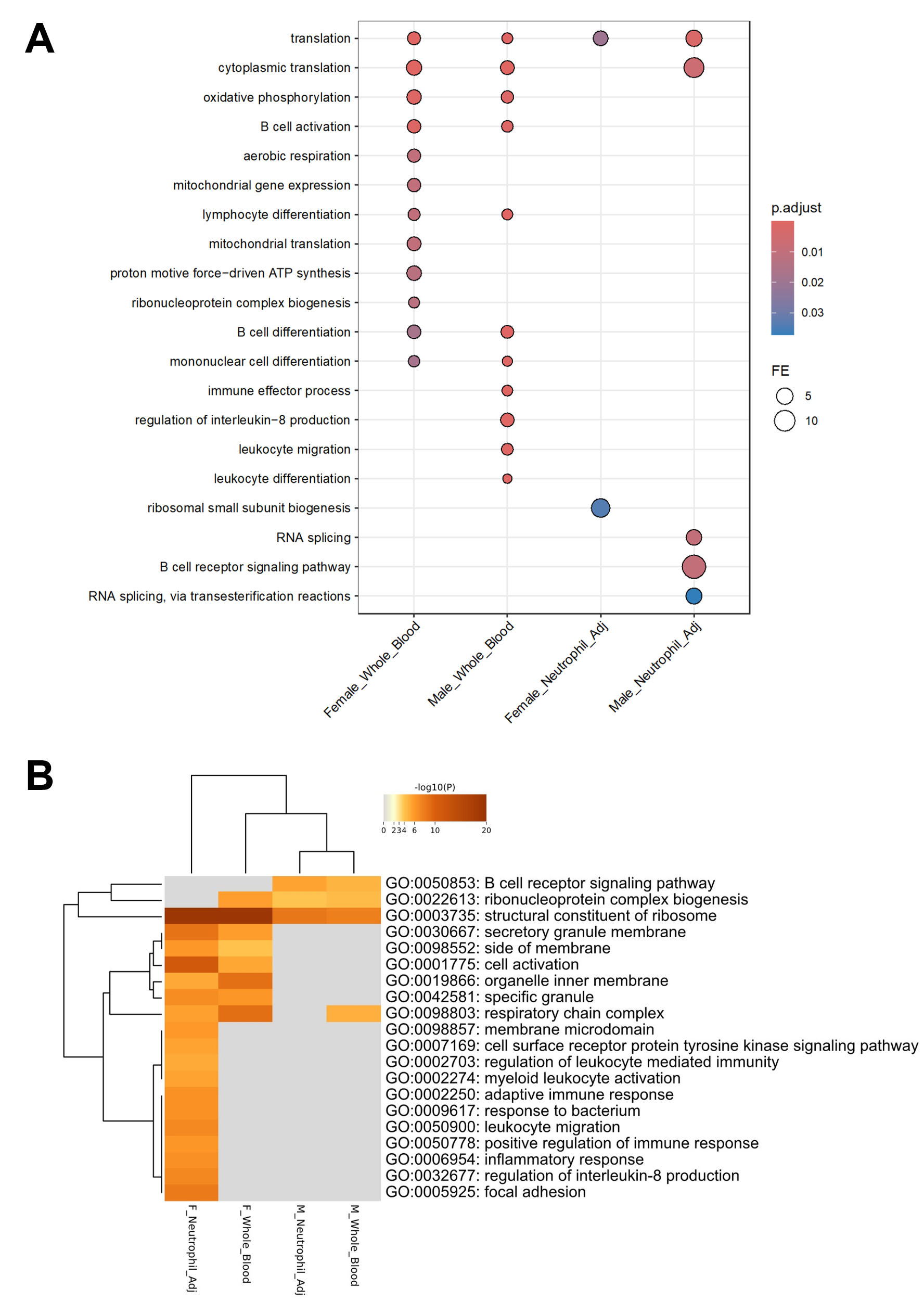


**Figure S4**. Two distinct databases for ontology analysis provide similar conclusions. Comparing biological pathways in AD, the influence of sex (women (n = 609) and men (n = 412)), and blood cell content. **A**: A dot-plot of significant DAVID GOTERM_BP_ALL gene ontology categories enriched in differentially expressed genes from whole blood transcriptomics in DE female whole blood, DE in male whole blood, DE in female neutrophil-adjusted blood, and DE in male neutrophil-adjusted blood. **B:** Metascape-based Gene ontology analysis of the same DE genes between controls and AD in whole blood or neutrophil-corrected data used in S4A. Abbreviations: Neutrophil_Adj, Neutrophil-adjusted data.


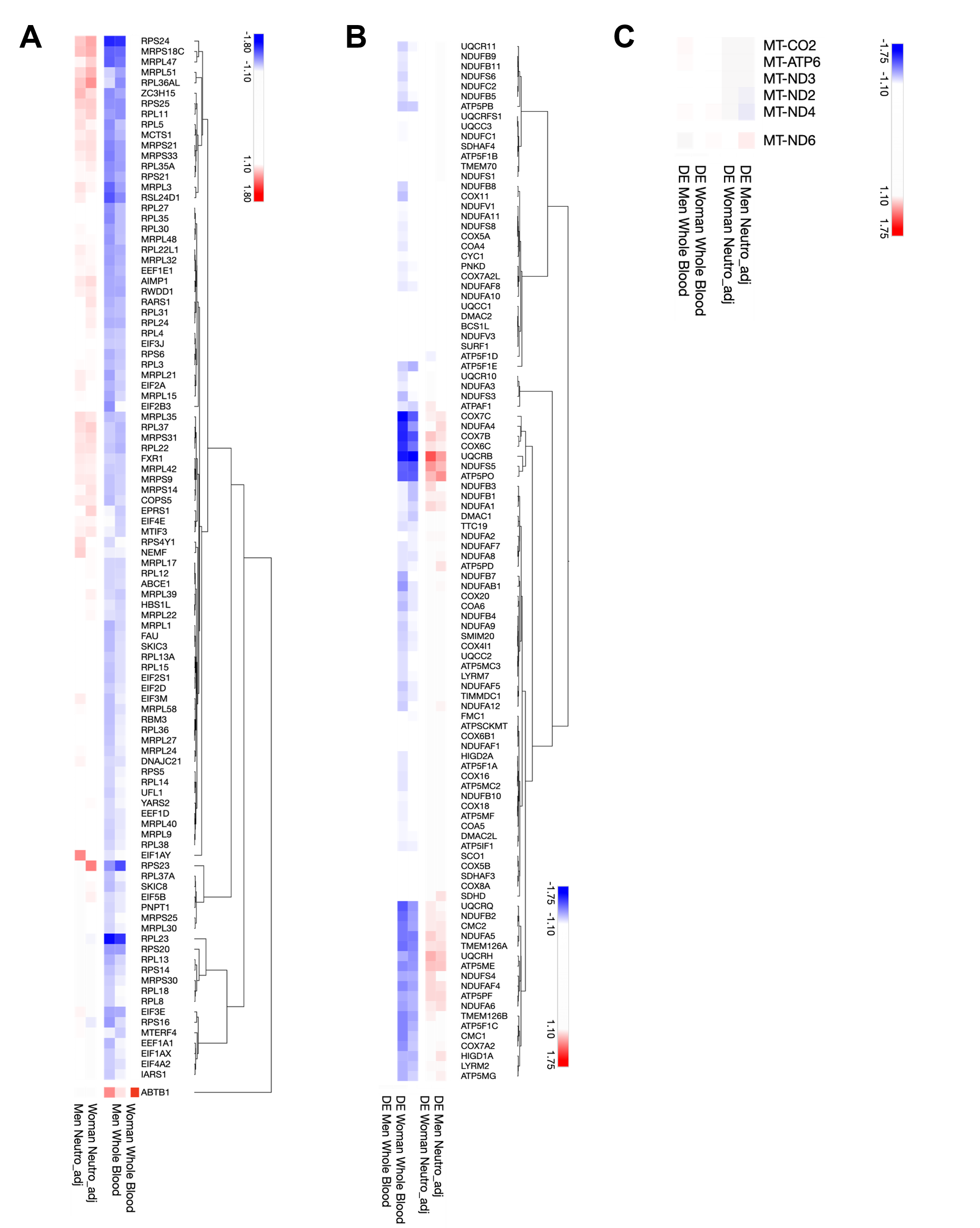


**Figure S5**. Using the gene-sets identified by DAVID (ribosomal) and the Broad Institute MitoCarta database, we replotted the DE values as heatmaps (Ribosomal and mitochondrial translation genes of core oxidative phosphorylation (OXPHOS) genes and finally genes encoded by the mitochondrial DNA (mtDNA). Each plot shows DE in male whole blood, female whole blood, male neutrophil-adjusted blood, and female neutrophil-adjusted blood. **A:** Ribosomal and mitochondrial translation transcripts. Both mitochondrial and protein translation expression were significantly regulated in AD but influenced by whole blood cell content and data processing. **B:** Nuclear-encoded OXPHOS genes. The directionality of the mitochondrial pathway expression is dependent on cell subtype influences. **C:** mtDNA genes. We find no evidence of altered mtDNA-encoded transcript expression in AD blood. Abbreviations: DE, Differential expression; OXPHOS, oxidative phosphorylation; mtDNA, mitochondrial DNA; Neutro_adj, neutrophil-adjusted blood.


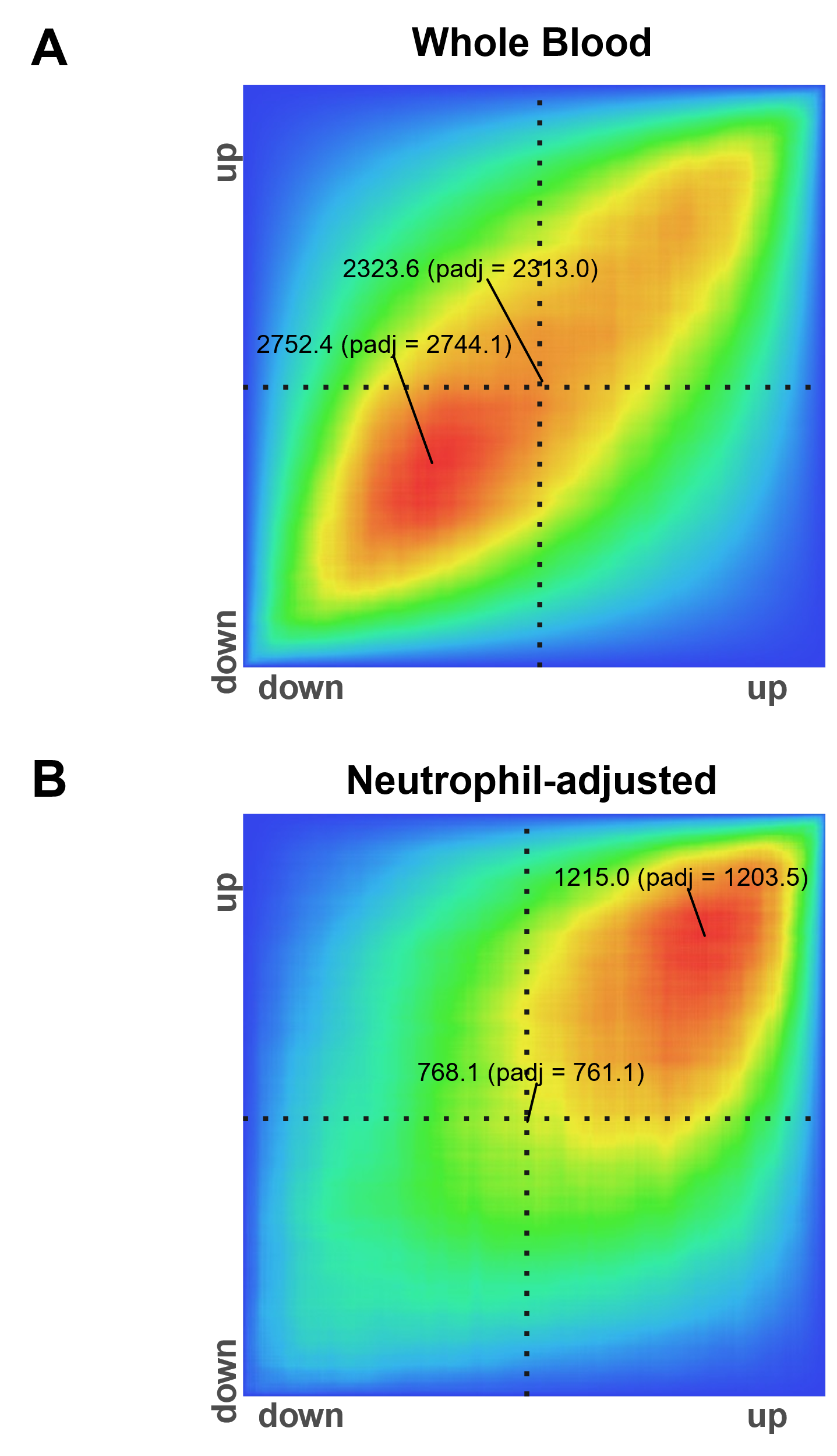


**Figure S6**. Examination of the consistency of differential gene expression (AD vs CTL) in men versus women, using the RedRibbon R Package. This ranking order method, relying on the hypergeometric distribution, allows us to directly compare the difference in gene expression in women (Control vs AD) with the difference in gene expression in men (Control vs AD), without applying a hard threshold. This is a more sensitive approach for evaluating whether two groups show consistency in biological response (compared with hard thresholding and Venn diagram-based membership analysis). **A:** GeneTitan whole blood gene expression corrected for technical variance and **B:** GeneTitan Neutrophil and technical-adjusted gene expression (See methods). Note that when we adjust for bias in neutrophil content, we flip the directionality of the (~same) AD-regulated genes (See Figure S7).


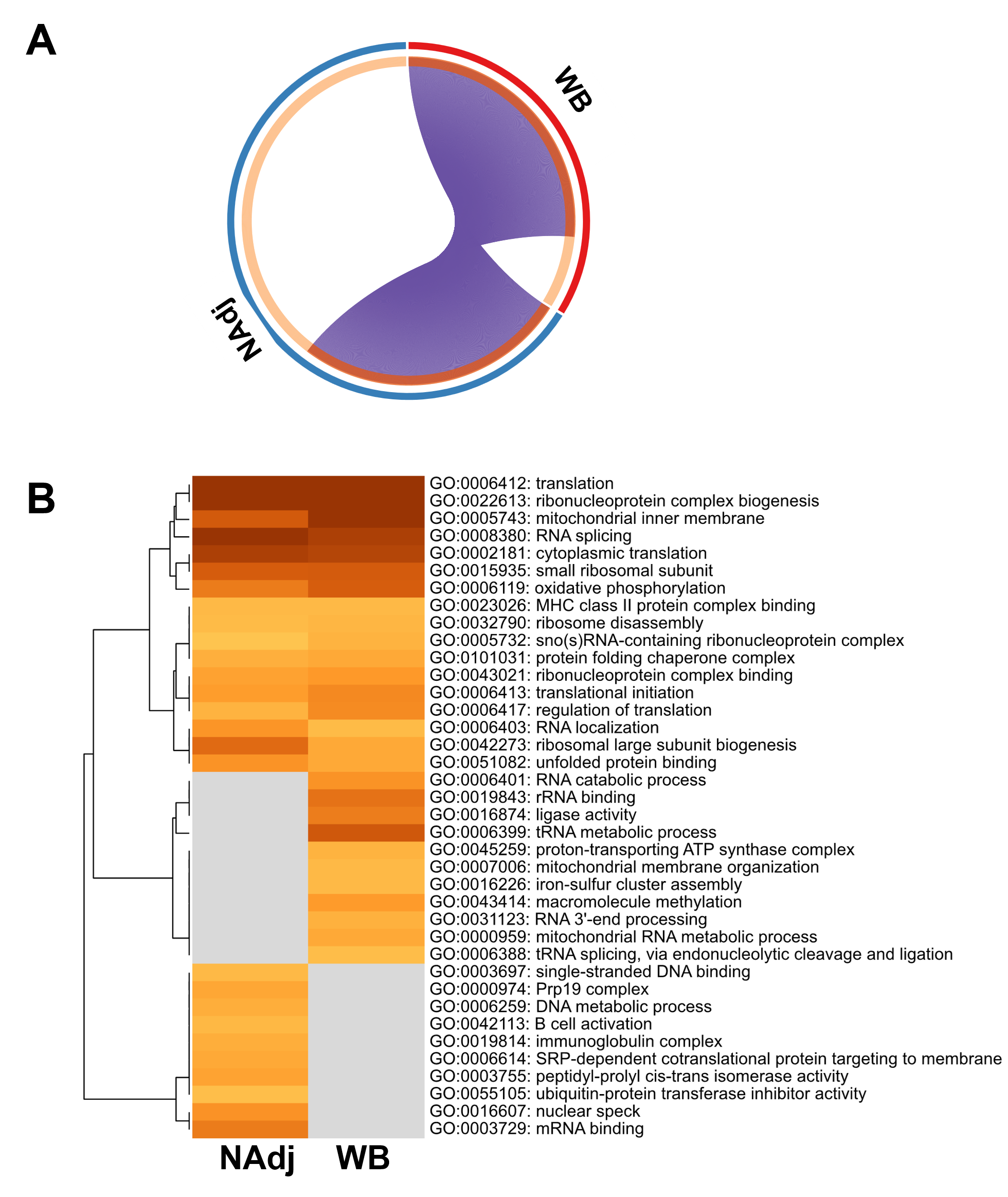


**Figure S7**. Examination of the consistency of differential gene expression (AD vs CTL) in men versus women, using the RedRibbon R Package. Genes from the highly consistent significant hot-spots (men vs women) for both transcriptomic data were profiled for shared biological pathways (using Metascape; a total of 3,043 genes with less than 10% difference in delta gene expression (control vs AD). **A:** Note that most genes consistently differentially expressed between Control and AD in men and women in whole blood were also consistently regulated in the Nadj data, but with distinct (See Figure S5) directionality. **B:** Shared pathways, regardless of data processing included protein translation, RNA splicing and some components of the mitochondria – including oxidative phosphorylation (again note the direction of differential switches and thus it can not be deemed a reliable biological feature of AD blood) while novel immunological features emerge in the NAdj data only (See main results and discussion). Abbreviations: AD, Alzheimer’s disease; CTL, Control; NAdj, neutrophil-adjusted; WB, Whole blood.


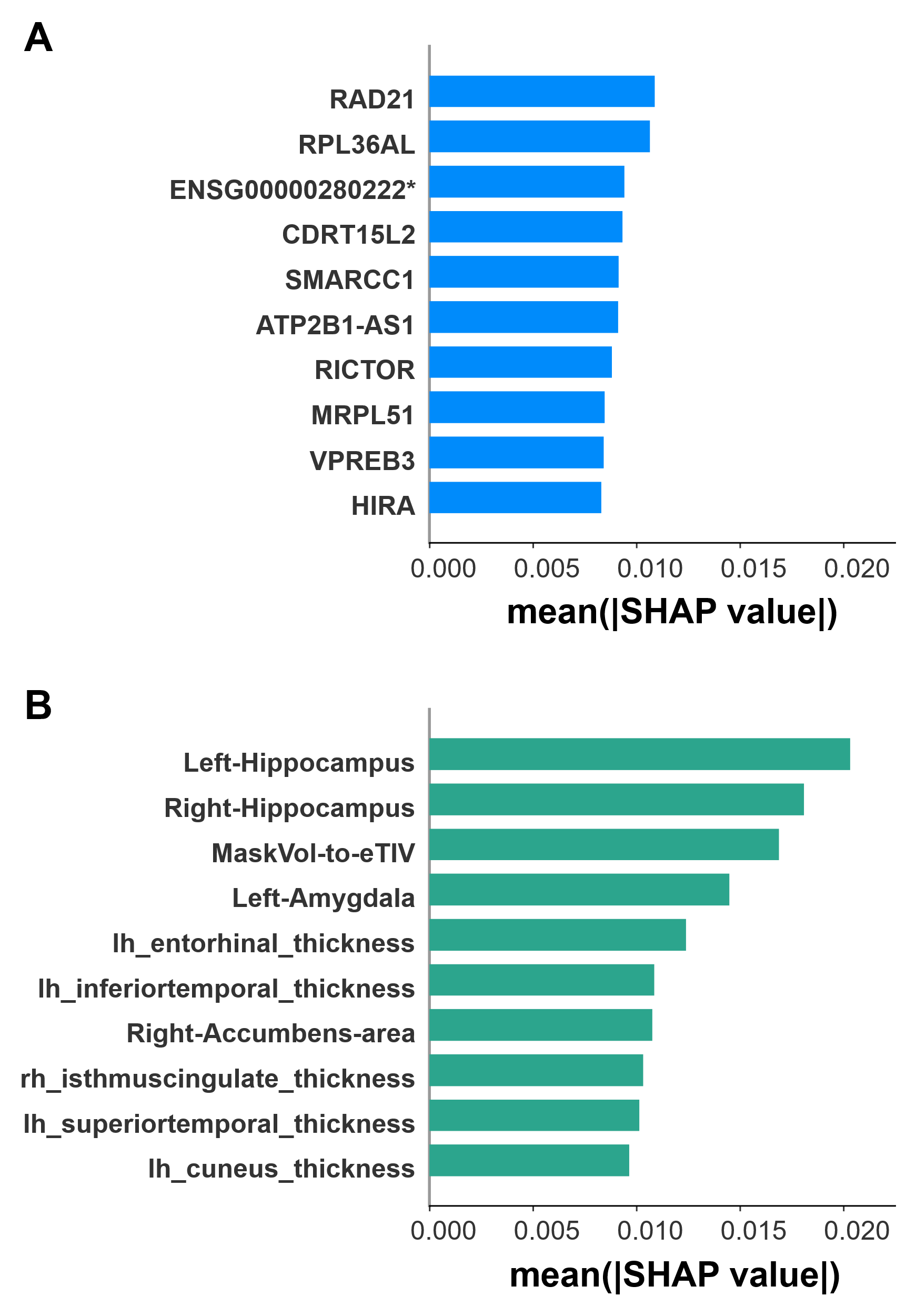


**Figure S8**. Assessment of the contribution of the top-ranked features to model performance. Shapley feature importance values were computed for input features for the best neutrophil-adjusted transcriptomics-MRI fusion models evaluated using 50 iterations of 5-fold stratified cross-validation (n=162; AD: 81, CTL: 81). Features are ordered by absolute mean Shapley value. **A:** Top 10 Transcriptomics features (blue). **B:** Top 10 MRI features (dark green). Abbreviations: SHAP, Shapley; MRI, Magnetic Resonance Imaging features extracted using Freesurfer 6.0. *ENSG00000280222 is a TEC (To Be Experimentally Confirmed) transcript, not associated with a gene symbol at the time of analysis.
